# AQP1 modulates tendon stem/progenitor cells senescence during tendon aging

**DOI:** 10.1038/s41419-020-2386-3

**Published:** 2020-03-18

**Authors:** Minhao Chen, Yingjuan Li, Longfei Xiao, Guangchun Dai, Panpan Lu, Youhua Wang, Yunfeng Rui

**Affiliations:** 1 Department of Orthopaedics, Zhongda Hospital, School of Medicine, Southeast University, Nanjing, 210009 Jiangsu China; 2 Orthopaedic Trauma Institute (OTI), Southeast University, Nanjing, 210009 Jiangsu China; 3 Trauma Center, Zhongda Hospital, School of Medicine, Southeast University, Nanjing, 210009 Jiangsu China; 4 China Orthopedic Regenerative Medicine Group, Hangzhou, 310000 Zhejiang China; 5 Department of Geriatrics, Zhongda Hospital, School of Medicine, Southeast University, Nanjing, 210009 Jiangsu China; 6 Department of Orthopaedics, Affiliated Hospital of Nantong University, Nantong, 226001 Jiangsu China

**Keywords:** Senescence, Ageing

## Abstract

The link between tendon stem/progenitor cells (TSPCs) senescence and tendon aging has been well recognized. However, the cellular and molecular mechanisms of TSPCs senescence are still not fully understood. In present study, we investigated the role of Aquaporin 1 (AQP1) in TSPCs senescence. We showed that AQP1 expression declines with age during tendon aging. In aged TSPCs, overexpression of AQP1 significantly attenuated TSPCs senescence. In addition, AQP1 overexpression also restored the age-related dysfunction of self-renewal, migration and tenogenic differentiation. Furthermore, we demonstrated that the JAK-STAT signaling pathway is activated in aged TSPCs, and AQP1 overexpression inhibited the JAK-STAT signaling pathway activation which indicated that AQP1 attenuates senescence and age-related dysfunction of TSPCs through the repression of JAK−STAT signaling pathway. Taken together, our findings demonstrated the critical role of AQP1 in the regulation of TSPCs senescence and provided a novel target for antagonizing tendon aging.

## Introduction

Tendon aging is a major risk factor for tendon disorder, such as chronic pain, tendon rapture and limited mobility^1,2^. With advancing age, the structural and functional properties of tendon have changed; tendon becomes more prone to degeneration and subsequent injury^3^. With existing medical treatments, it is still difficult to restore the original property of injured tendons. Previous studies have demonstrated that tendon aging is closely associated with the functional changes of tendon stem/progenitor cells (TSPCs)^4,5^. TSPCs were first reported by Bi et al.^6^ and have been isolated from various species^7,8^. TSPCs express classical stem cell markers and typical tendon-lineage genes. In addition, TSPCs are capable of self-renewal, clonogenicity and multilineage differentiation^6^. Studies have demonstrated the vital role of TSPCs in tendon repair, regeneration and homeostasis maintaining^9,10^. However, TSPCs premature entry into senescence during tendon aging^4,11^, senescent TSPCs exhibit reduced self-renewal, migration and tenogenic differentiation capacity compared with young cells, and these age-related changes in TSPCs would impair tendon healing and regeneration capacity.

Aquaporins (AQPs) are a family of small water-transporting membrane proteins that transport water and small molecules for the maintenance of fluid homeostasis^12^. It has been extensively reported that AQPs are widely expressed in various tissues and associated with many physiological functions or diseases^13,14^. Fluid loss occurs in most tissues with age, and this loss is closely associated with altered expressions of AQPs^15^. Aquaporin 1 (AQP1) is one of the members of the AQPs family and has been reported to play a vital role in various biological and pathological events, such as proliferation, inflammation and migration^16^. Previous studies also indicated that AQP1 is involved in tissue aging. In aged skin tissue, AQP1 level was decreased, which might be correlated with aging-related skin dryness^17^. Bicakci et al.^18^ reported that AQP1 might play a vital role in the maintenance of water and electrolytes balance in aged heart tissue. In addition, recent studies have indicated that AQP1 is also involved in the regulation of stem cells. Graziano et al.^19^ reported that AQP1 was functionally involved in homeostasis and chondrogenic differentiation of adipose-derived mesenchymal stem cells. Meng et al.^20^ demonstrated that AQP1 could promote bone marrow mesenchymal stem cells migration through β-catenin and FAK pathways in a rat fracture model. Although studies have indicated the important role of AQP1 in tissue aging and regulation of stem cell, there were no studies focussing on the role of AQP1 in TSPCs senescence.

In the present study, we investigated the AQP1 expression profile of TSPCs isolated from rats at different ages. We demonstrated that AQP1 expression declines with age in TSPCs, and AQP1 plays a vital role in TSPCs senescence. Decreased AQP1 was associated with activation of JAK-STAT signaling pathways in aged TSPCs. Furthermore, overexpression of AQP1 restored the age-related reduction of self-renewal, migration and tenogenic differentiation in TSPCs. Our results collectively indicated that AQP1 could be an ideal target for antagonizing tendon aging.

## Results

### AQP1 expression declines with age in TSPCs

**Fig. 1:**
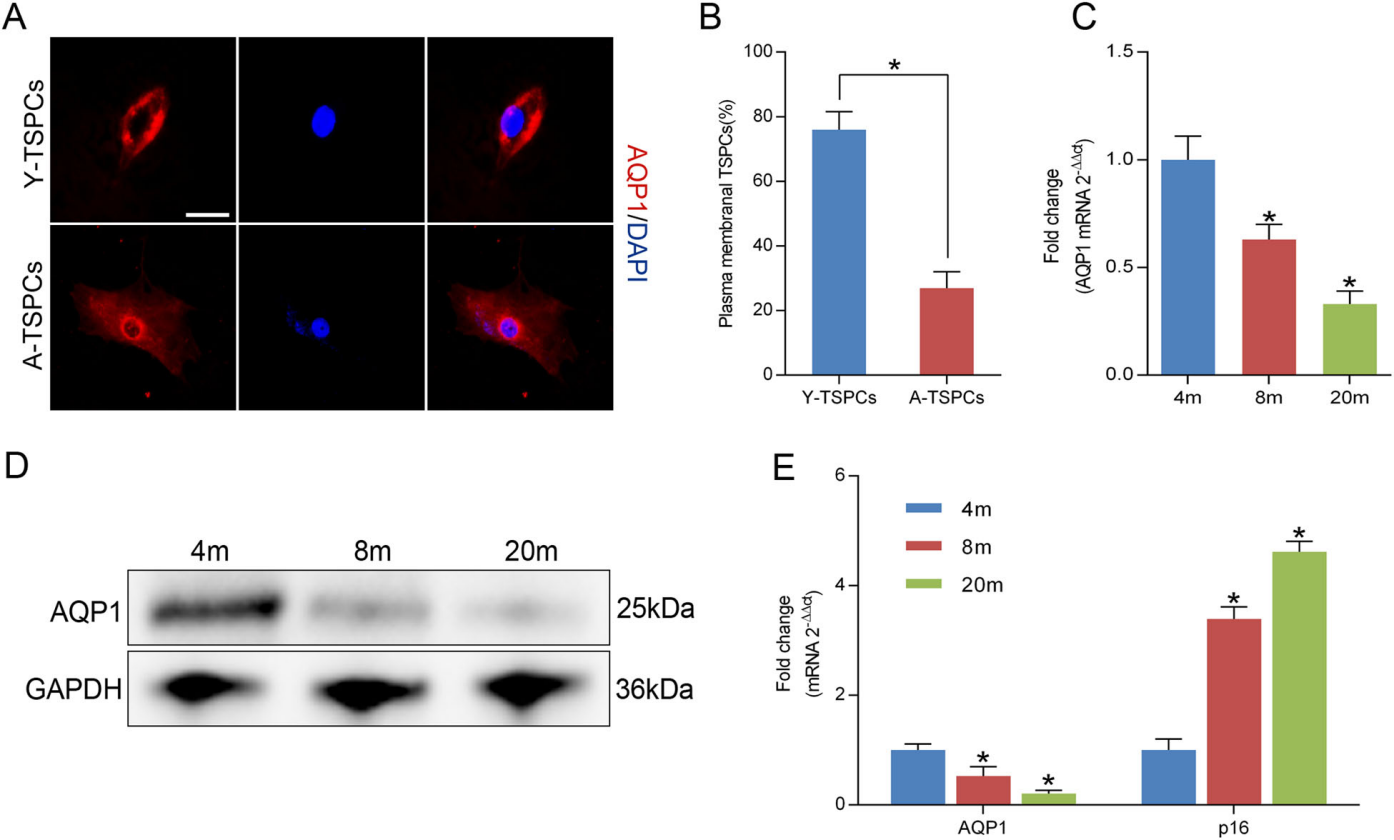
AQP1 expression is reduced in senescent TSPCs. **a** Immunofluorescence staining for AQP1 in TSPCs. Scale bar: 20 μm. **b** Quantitative analysis of cells with plasma membranal AQP1. **c** Relative mRNA levels of AQP1 in TSPCs were investigated by qRT-PCR. **d** Western blotting for the AQP1 protein levels in TSPCs isolated from 4-, 8-, and 20-month-old rats. **e** Inverse expressions of AQP1 and p16^INK4A^ were assessed by qRT-PCR. Values represent the mean ± SD. *P < 0.05, significantly different from the young group.

Immunofluorescence staining revealed that AQP1 is predominantly expressed in plasma membrane in young TSPCs (Y-TSPCs), whereas aged TSPCs (A-TSPCs) showed AQP1 distributed primarily within the nuclear membrane (Fig. 1a, b). AQP1 expression declined progressively during their progression from young (4 months) to aged (8–20 months) (Fig. 1c, d). Moreover, the mRNA level of senescence marker p16^INK4A^ was progressively increased in aged TSPCs, while the AQP1 mRNA level inversely correlated with p16^INK4A^ (Fig. 1e). The results indicated that AQP1 expression inversely correlates with the progression of TSPCs senescence.

### AQP1 regulates TSPCs senescence

**Fig. 2:**
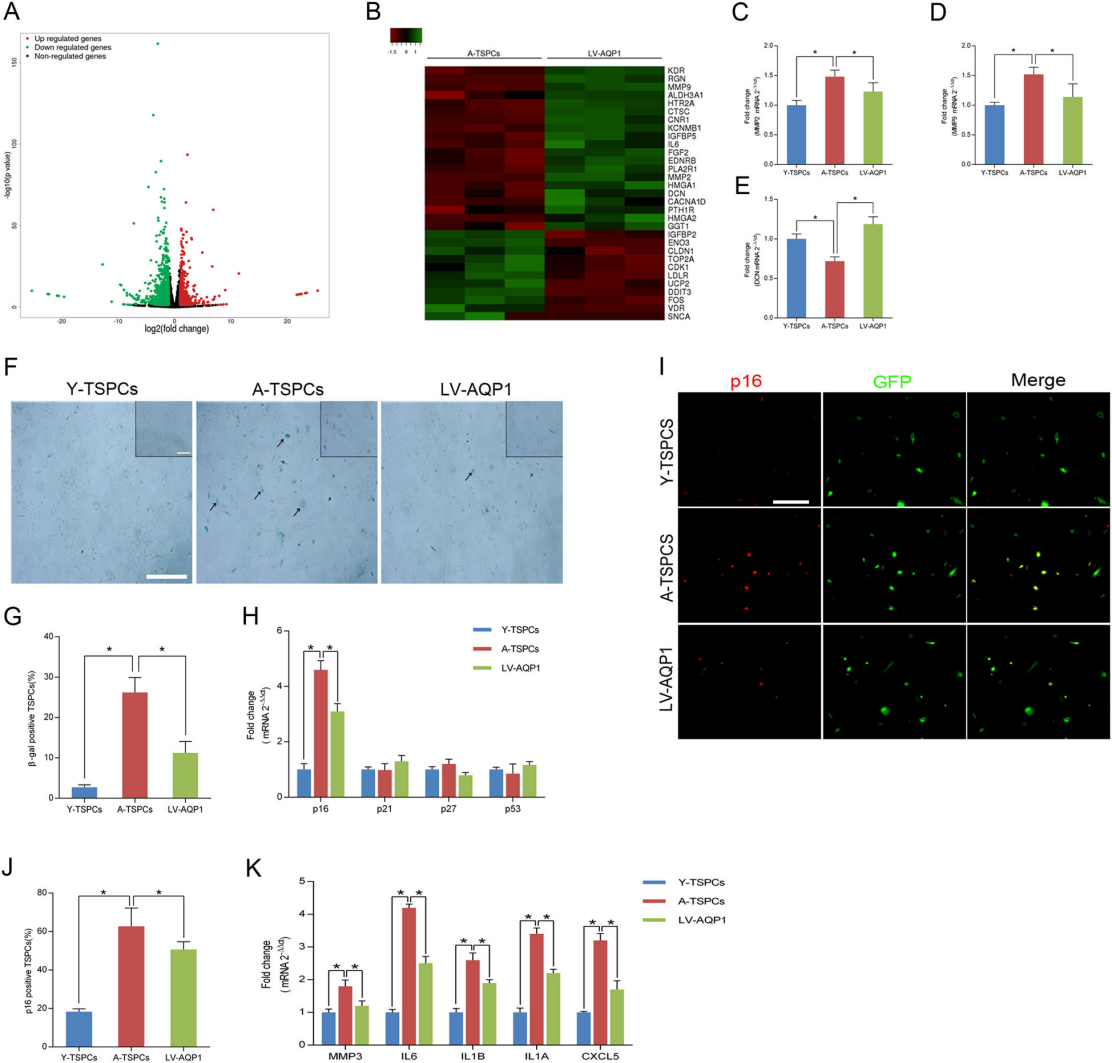
AQP1 regulates TSPCs senescence. **a** Volcano plot showed genes that were differentially expressed after LV-AQP1 transfection. **b** Heatmap showed the changes in the expression of aging-related genes in aged and AQP1-overexpressing aged TSPCs. **c**–**e** Expressions of genes involved in tendon aging-related genes (MMP2, MMP9, DCN) were assessed by qRT-PCR in TSPCs. **f** β-gal staining for the senescent cells in TSPCs. Scale bars: 200 μm (larger images); 100 μm (inset). **g** Quantitative analysis of β-gal-positive TSPCs. **h** qRT-PCR for the cell cycle regulators (p16^INK4A^, p21, p27, p53) levels in TSPCs. **i** Immunofluorescence staining for p16^INK4A^ in TSPCs. Scale bar: 50 μm. **j** Quantitative analysis of p16^INK4A^-positive cells. **k** qRT-PCR for senescence-associated secretory phenotype (SASP) genes (MMP3, IL6, IL1B, IL1A and CXCL5) expressions in TSPCs. Values represent the mean ± SD. *P < 0.05, significantly different from the young or aged group.

To examine the role of AQP1 in TSPCs senescence, aged cells were stably transduced with lentivirus encoding AQP1 (LV-AQP1). The transfection efficiency was detected by Western blotting (Fig. S1A). Microarray analysis showed that 1070 mRNAs were upregulated and 573 mRNAs were downregulated (fold change ≥ 2) after LV-AQP1 transfection (Fig. 2a and Fig. S1B–E). We then analyzed aging-related microarray data and found a marked change in the expression of genes involved in tendon aging, including MMP2, MMP9, DCN (Fig. 2b–e). Moreover, β-gal staining showed that LV-AQP1 treatment reduced the β-gal-positive senescent cells in aged TSPCs, which suggested its senescence-delaying potential (Fig. 2f, g). This was coupled with a significant repression of p16^INK4A^ in aged TSPCs (Fig. 2h). The p16^INK4A^ downregulation in AQP1-overexpressing aged TSPCs was also confirmed by immunofluorescence staining (Fig. 2i, j). Besides, we investigated the expression of senescence-associated secretory phenotype (SASP) genes, including MMP3, IL6, IL1B, IL1A and CXCL5, and we found that these SASP genes were highly upregulated in aged TSPCs, while LV-AQP1 treatment reduced SASP expression (Fig. 2k).

To investigate the role of AQP1 in TSPCs senescence further, we used AQP1-siRNA to knockdown AQP1 in young TSPCs (Fig. S2A). The results showed that AQP1 knockdown increased the β-gal-positive senescent cells, as well as the expression of p16^INK4A^ in young TSPCs (Fig. S2B–D). Moreover, AQP1 knockdown also induced the dysfunction of self-renewal, migration and tenogenic differentiation in young TSPCs (Fig. S2E–J). These results indicated that AQP1 plays a vital role in TSPCs senescence.

### Overexpression of AQP1 in aged TSPCs promotes their self-renewal capacity

**Fig. 3:**
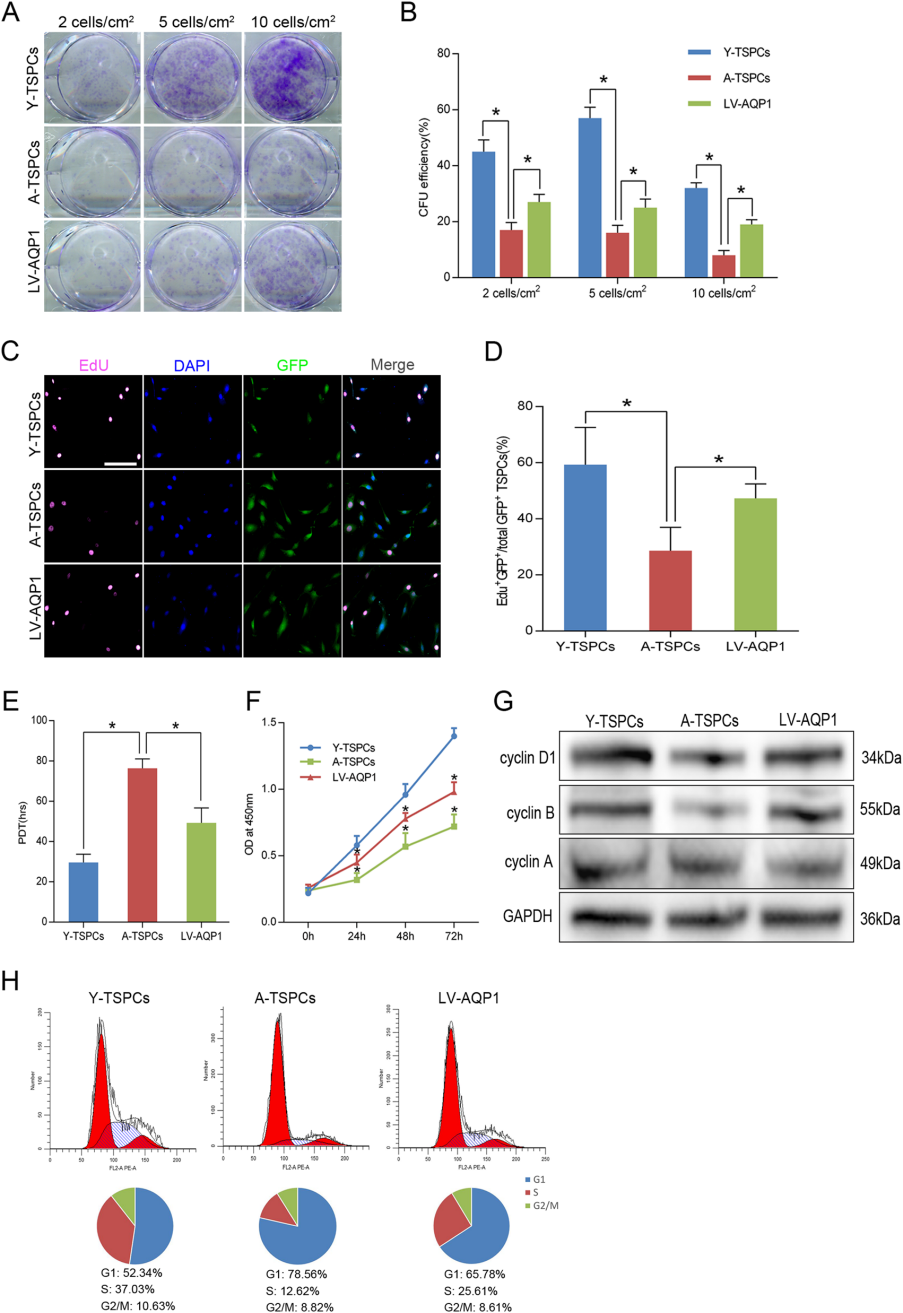
Overexpression of AQP1 in aged TSPCs restores their self-renewal capacity. **a** Colony-forming unit (CFU) assay was performed at three densities, crystal violet-stained colonies at day 14. **b** CFU efficiency. **c**, **d** Representative images for EdU incorporation in TSPCs and quantification of EdU^+^ GFP^+^/total GFP^+^ cells. Scale bar: 20 μm. **e**, **f** Proliferation rate of TSPCs was measured by population doubling time (PDT) assay and CCK-8 assay. **g** Western blotting for the cell-cycle-related proteins expressions in TSPCs. **h** The cell cycle distribution of TSPCs was measured by flow cytometry. The pie charts summarizing cell fractions in the G1, S and G2/M phases of the cell cycle. Values represent the mean ± SD. *P < 0.05, significantly different from the young or aged group.

Given the ability of AQP1 to attenuate TSPCs senescence, we tested whether overexpression of AQP1 affects the self-renewal capacity of senescent TSPCs. Treatment with LV-AQP1 significantly increased the colony number and CFU efficiency in the aged TSPCs (Fig. 3a, b). We also observed that LV-AQP1 treatment notably enhanced the proliferative potential of aged TSPCs, as evidenced by 5-ethynyl-2′-deoxyuridine (EdU) detection, population doubling time (PDT) and CCK-8 assay (Fig. 3c–f). We also found a marked change in the expression of genes involved in proliferation after transfection by gene ontology (GO) analysis (Table S1). To explore the molecular changes in AQP1-overexpressing aged TSPCs, we performed cell cycle analyses of TSPCs (Fig. 3g, h); as compared to young TSPCs, the expressions of cyclin D1 and cyclin B were significantly reduced in aged TSPCs, and the proportion of aged TSPCs was also increased in G1 phase. Of note, the accumulation of aged TSPCs at G1 phase and reduced levels of cyclins D1 and cyclin B were blocked after LV-AQP1 treatment. Collectively, these results indicated the role of AQP1 in promoting self-renewal capacity of aged TSPCs.

### LV-AQP1 treatment restores the migration deficit of aged TSPCs

**Fig. 4:**
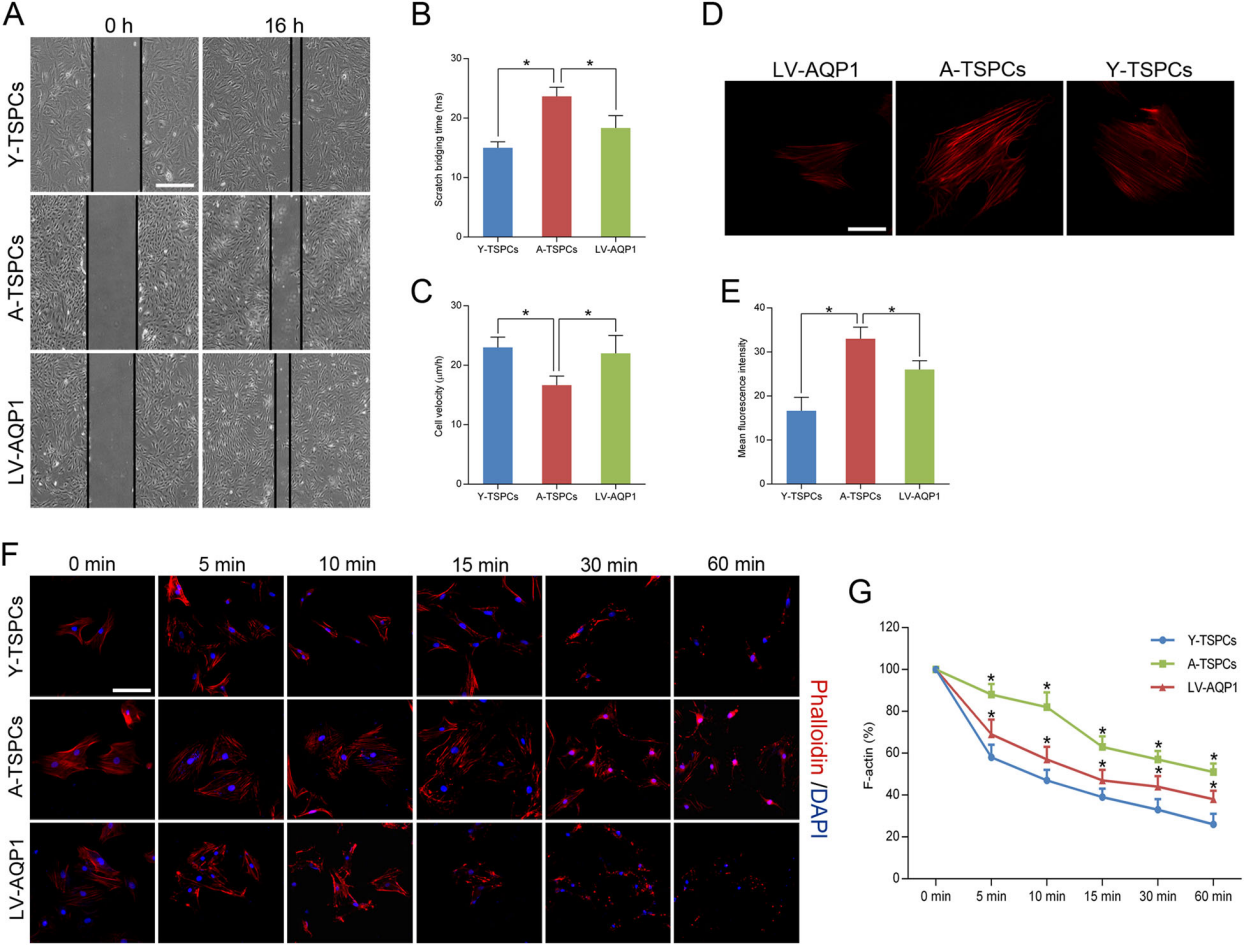
AQP1 overexpression restores the migration deficit of aged TSPCs. **a** Representative scratch assay of young, aged and AQP1-overexpressing aged TSPCs. Scale bar: 200 μm. **b**, **c** Quantification of scratch bridging time and cell velocity. **d** Phalloidin staining for TSPCs. Scale bar: 20 μm. **e** F-actin mean fluorescence intensity in young, aged and AQP1-overexpressing aged TSPCs. **f** Representative images of phalloidin labeling at each time point in TSPCs after latrunculin A treatment. DAPI was used for nuclear staining. Scale bar: 50 μm. **g** Quantification of F-actin amount upon latrunculin A treatment. The F-actin content at the beginning (0 min) was set to 100%. Values represent the mean ± SD. *P < 0.05, significantly different from the young or aged group.

To investigate the role of AQP1 in TSPCs migration, we performed a scratch assay; the result revealed a decelerated migration and longer scratch bridging time in the aged TSPCs while LV-AQP1 restored the age-related migration deficit (Fig. 4a–c).

Previous studies showed that actin cytoskeleton organization and the rate of actin turnover are vital for cell migration^21^. Here, we performed phalloidin staining and compared the actin dynamics by treating with latrunculin A in a time-dependent manner. We found a bigger cell area and a higher F-actin content in aged TSPCs, while LV-AQP1 treatment did not affect the cell area of aged TSPCs (Fig. 4d). Moreover, AQP1 overexpression significantly reduced the F-actin content in aged TSPCs, which indicated the improved actin turnover of aged TSPCs (Fig. 4e–g). Using RNA-seq, we also found marked changes in the expression of genes involved in migration and actin dynamics in AQP1-overexpressing TSPCs (Table S2). These findings suggested that LV-AQP1 treatment improves migration and actin dynamics of aged TSPCs.

### AQP1 overexpression promotes tenogenic differentiation of aged TSPCs

**Fig. 5:**
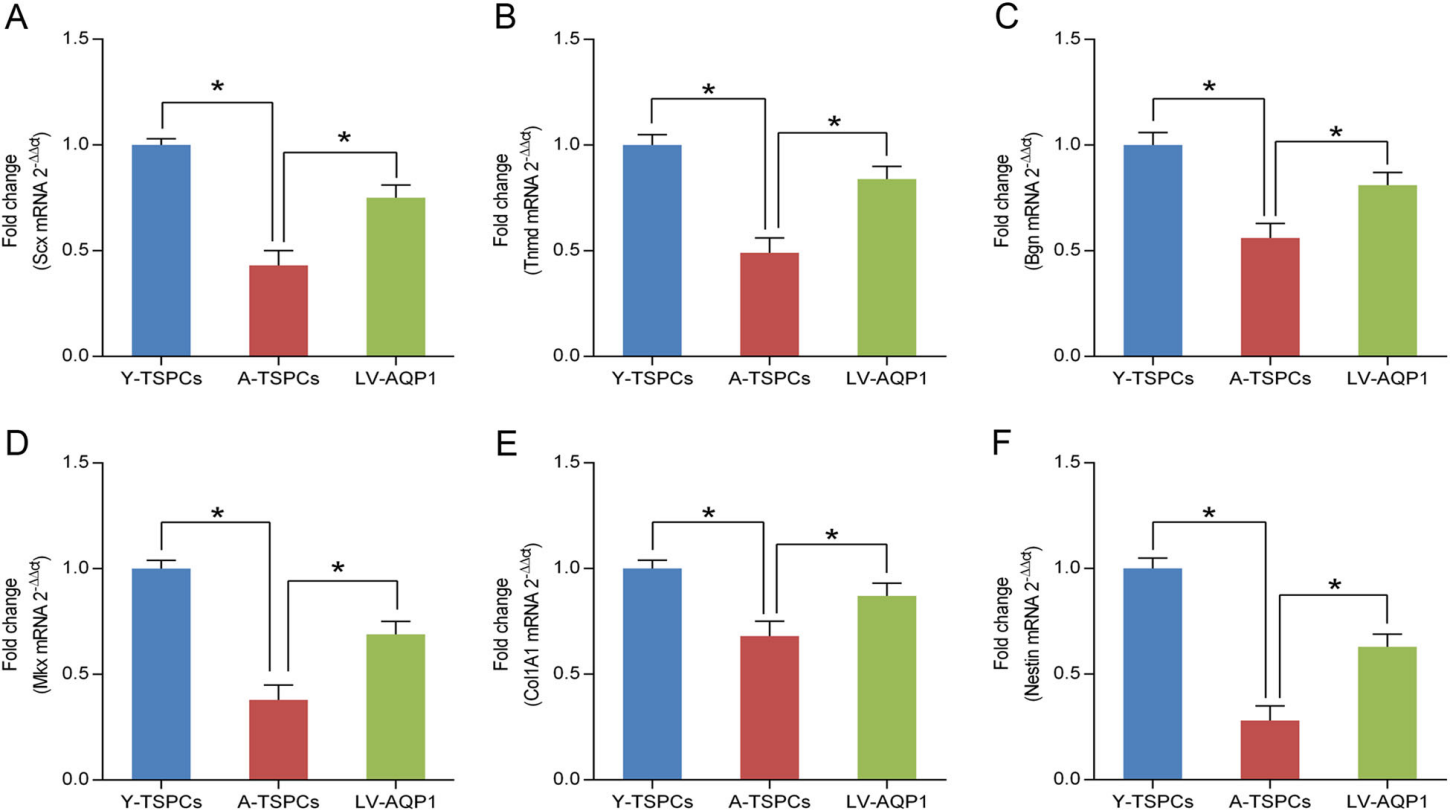
AQP1 regulates and promotes the tendon-related genes expression. **a**–**f** Relative mRNA levels of tendon-related genes (Scx, Tnmd, Bgn, Mkx, Col1A1 and Nestin) in young, aged and AQP1-overexpressing aged TSPCs were investigated by qRT-PCR. Values represent the mean ± SD. *P < 0.05, significantly different from the young or aged group.

To study the role of AQP1 in the tenogenic differentiation capacity of TSPCs, we investigated the key tendon-related markers of TSPCs, including Scx (scleraxis), Tnmd (tenomodulin), Bgn (Biglycan), Mkx (mohawk), Col1A1 (collagen type I a 1 chain), and Nestin. The results showed that these markers were significantly reduced in aged TSPCs, which suggested that aging suppresses the key tendon-related markers expression of TSPCs (Fig. 5a–f). Meanwhile, AQP1 overexpression could rescue the decreased levels of the tendon-related markers. These results showed a critical role of AQP1 in tenogenic differentiation.

### AQP1 attenuates TSPCs senescence through JAK-STAT pathway

**Fig. 6:**
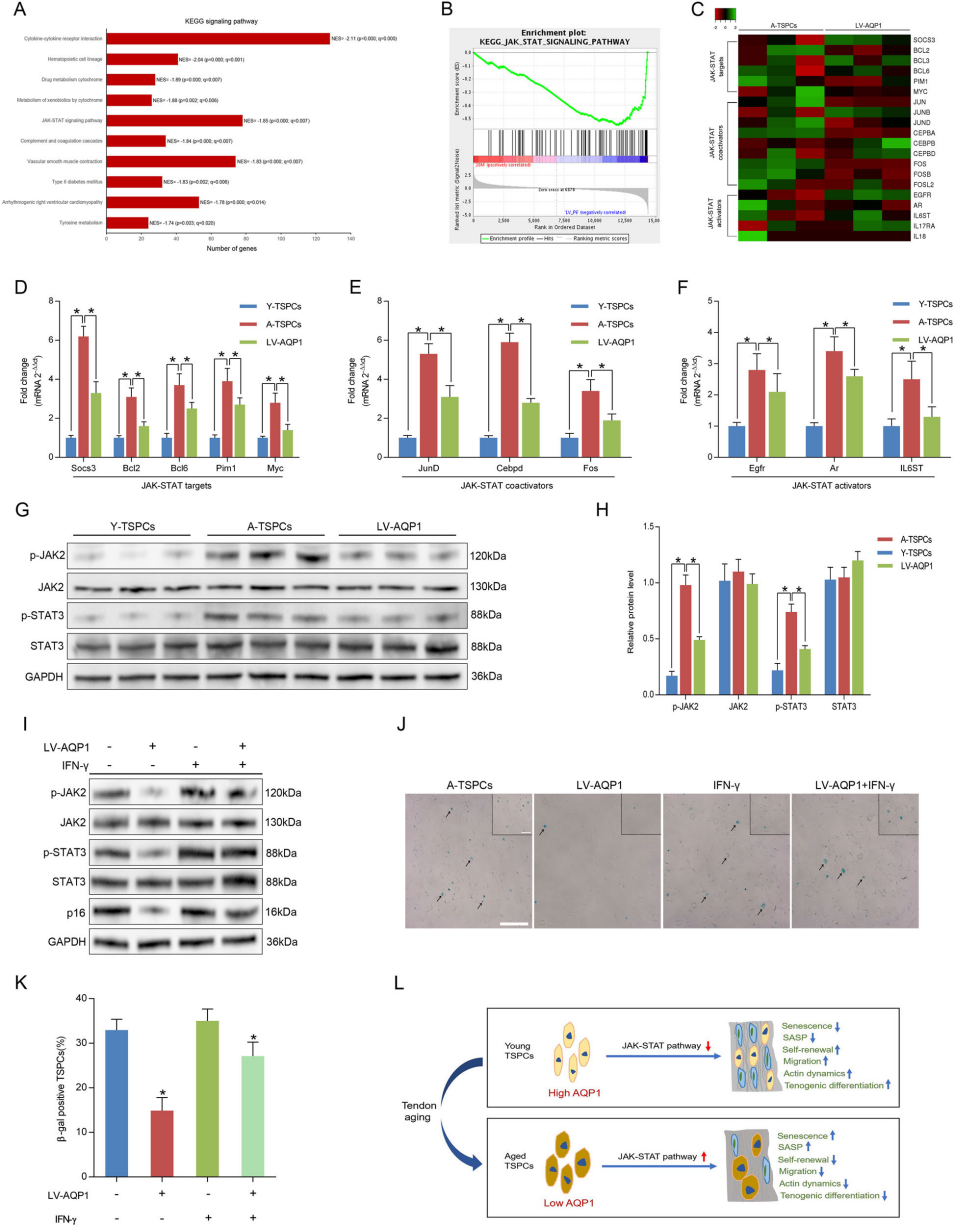
AQP1 regulates TSPCs senescence through JAK-STAT pathway. **a** The GSEA KEGG analysis revealed the top ten significant enriched signaling pathways in AQP1-overexpressing aged TSPCs. **b** GSEA plots showed a gene set related to JAK-STAT pathway. **c** Heatmap showed the changes in the expression of JAK-STAT targets, coactivators and activators in aged and AQP1-overexpressing aged TSPCs. **d**–**f** Relative mRNA levels of JAK-STAT targets (Socs3, Bcl2, Bcl6, Pim1 and Myc), coactivators (JunD, Cebpd and Fos) and activators (Egfr, Ar and IL6ST) in TSPCs were validated by qRT-PCR. **g**, **h** Western blotting for the p-JAK2, JAK2, p-STAT3 and STAT3 protein levels in young, aged and AQP1-overexpressing aged TSPCs. **i** Aged TSPCs were transfected with or without LV-AQP1 and then stimulated with IFN-γ at 100 ng/ml for 4 h. The expressions of p-JAK2, JAK2, p-STAT3, STAT3 and p16^INK4A^ were investigated by western blotting. **j**, **k** β-gal staining for the senescent cells in aged TSPCs upon LV-AQP1 and/or IFN-γ (100 ng/ml, 24 h) treatment. Scale bars: 200 μm (larger images); 100 μm (inset). **l** Model by which AQP1 modulates senescence during tendon aging. In young TSPCs, AQP1 inhibits the activation of JAK-STAT signaling pathway to maintain the normal cell function. In aged TSPCs, reduction of AQP1 causes JAK-STAT activation, leading to impaired cell function. Values represent the mean ± SD. *P < 0.05, significantly different from the young or aged group.

To identify signaling pathways and downstream targets regulated by AQP1 in aged TSPCs, we analyzed gene sets using the Kyoto encyclopedia of genes and genomes (KEGG) suite in gene set enrichment analysis (GSEA) to identify enriched signaling pathways in aged and AQP1-overexpressing TSPCs. The GSEA KEGG analysis showed the significant enriched signaling pathways on the basis of normalized enrichment score (NES) in aged and AQP1-overexpressing aged TSPCs (Fig. 6a and Fig. S3A). Notably, JAK-STAT signaling pathway has been reported to be linked to TSPCs function^22^, as well as the cellular senescence^23,24^. In addition, GSEA revealed that AQP1 is negatively correlated with JAK-STAT signaling pathway (Fig. 6b). The heatmap showed the gene set involved in JAK-STAT signaling (Fig. 6c and Fig. S3B). We then validated the expression of genes involved in JAK-STAT signaling pathway by qRT-PCR; the result showed that JAK-STAT targets (Socs3, Bcl2, Bcl6, Pim1 and Myc), JAK-STAT coactivators (JunD, Cebpd and Fos) and JAK-STAT activators (Egfr, Ar and IL6ST) were significantly increased in aged TSPCs as compared to young TSPCs, while AQP1 overexpression could rescue the increased level of these genes in aged TSPCs (Fig. 6d–f). Furthermore, we also found that the protein levels of p-JAK2 and p-STAT3 were notably higher in aged TSPCs, which further suggested the activation of JAK-STAT signaling. Of note, LV-AQP1 treatment inhibited the phosphorylation of JAK2 and STAT3 (Fig. 6g, h). We next treated aged TSPCs with interferon-γ (IFN-γ), an activating cytokine of JAK-STAT signaling pathway^25^; the results showed that IFN-γ treatment abolished LV-AQP1-induced inhibition of p16^INK4A^ expression, as well as the number of β-gal-positive senescent cells (Fig. 6i–k). To further investigate the association between AQP1 and JAK/STAT signaling in TSPCs senescence, we use the JAK-STAT signaling pathway inhibitor AG490 to treat young TSPCs. Notably, AG490 treatment also reversed the AQP1-siRNA-induced senescence of TSPCs (Fig. S4). Collectively, our findings suggested that AQP1 attenuates TSPCs senescence, at least in part, due to the repression of JAK-STAT signaling during tendon aging.

## Discussion

Tendon aging has been linked to the process of TSPCs senescence. Here, we reported for the first time that AQP1 expression is closely correlated with TSPCs senescence and plays a vital role in the resistance to senescence. The flow of fluid across cell membranes is essential for the physiology of organisms, and the membrane permeability to fluid is a function of properties of AQPs in cell membranes^26^. Altered expressions or functions of AQPs lead to fluid loss, and this loss is closely associated with cell aging^15^. Furthermore, AQPs can also facilitate the diffusion of reactive oxygen species, which lead to cellular alterations related to cell aging^15,27^. Studies have showed that functional AQP1 is inserted into the plasma membrane^28,29^. Localization of AQP1 can be regulated by osmotic stress-mediated calcium signal; a sustained calcium signal is necessary for continued AQP1 plasma membrane localization^26^. Interestingly, our results indicated that the plasma membrane localization of AQP1 is decreased, whereas the nuclear membrane staining is dramatically increased in aged TSPCs. We speculated that the abnormal translocation of AQP1 may be associated with altered calcium signal in aged TSPCs. Furthermore, we showed that the expression of AQP1 declines with age in TSPCs, overexpression of AQP1 attenuated cellular senescence in aged TSPCs. AQP1 regulates TSPCs senescence partially dependent on the cell cycle inhibitor p16^INK4A^; p16^INK4A^ could be an ideal marker for TSPCs senescence^4^. Senescence represents a state of permanent cell cycle arrest; senescent cells secrete a variety of factors collectively known as SASP, which further reinforce the arrest in a paracrine manner^30,31^. SASP expression varies according to different environments and different cell types^32,33^. In this study, we reported that SASP genes were significantly increased in aged TSPCs, while AQP1 overexpression attenuated SASP expression. Collectively, these results indicate that AQP1 plays a critical role of antisenescence in TSPCs.

TSPCs differ in their ability to self-renew and multilineage differentiation to tenocytes; TSPCs self-renew to maintain a pool of healthy stem cells^34^. However, during tendon aging, the TSPCs pool becomes exhausted and may eventually lead TSPCs to enter into senescence^4^. Previous studies showed that the clonogenicity, PDT and cell numbers of aged TSPCs are significantly lower than young TSPCs^4,35^. In the present study, we also showed an attenuated self-renewal ability in aged TSPCs, which is in accordance with the depletion of TSPCs pool in aged tendon as mentioned above. AQP1 overexpression decreased the PDT and increased clonogenicity and proliferative rate of aged TSPCs, which supported a vital role of AQP1 in regulating self-renewal ability of TSPCs. Furthermore, we also found that AQP1 overexpression promoted the cell cycle progression in aged TSPCs; the result is consistent with the role of AQP1 in maintaining cyclins expressions and in G1/S transition in other cell types^36,37^.

Stem cell migration is required for tissue repair; migration of TSPCs to the injury site is important for tendon-healing process^38^. Previous studies have indicated the attenuated migratory capacity and actin dynamics in aged TSPCs^4,39^. Here, we demonstrated that AQP1 significantly promotes aged TSPCs migration, which is in line with a positive effect of AQP1 in stem cell migration as several studies reported^20,40^. Actin cytoskeleton and its dynamics are vital for cell migration^21^; we next performed latrunculin A experiments to compare the actin dynamics of TSPCs; latrunculin A inhibits actin polymerization and thereby disrupts the turnover of actin filaments^4,39^. Our data suggested that AQP1 overexpression significantly improves the actin turnover of aged TSPCs. Thus, the enhanced migratory capacity of AQP1-overexpressing TSPCs may be associated with the improved actin dynamics, and the ability of AQP1 to promote TSPCs migration may facilitate tendon repair.

Accumulating evidence suggested that the expressions of key tendon-related markers are significantly reduced in aged TSPCs^11,41^. Consistent with previous studies, our study also showed that the expressions of key tendon-related markers were reduced in aged TSPCs, which suggested the impaired tenogenic differentiation ability. Collagen type I is the main component of the extracellular matrix of tendon and maintains the normal structure^42^. Mkx plays a critical role in tendon development by regulating collagen type I^43^. It has been reported that Bgn, Scx and Tnmd act as important regulators of tenogenic differentiation and tendon healing^11^. Yin et al.^44^ reported that Nestin-positive TSPCs showed a superior tenogenic capacity compared with Nestin-negative TSPCs. In addition, AQP1 overexpression rescued the decreased level of these markers in aged TSPCs, which suggested a requirement of AQP1 in regulating tenogenic differentiation.

JAK-STAT signaling pathway has been reported to be involved in stem cell senescence. Price et al.^23^ reported that JAK-STAT pathway was activated in aged satellite cells that significantly contributed myogenic commitment and results in their regenerative deficiency. Ji et al.^24^ showed activation in senescent bone marrow mesenchymal stem cells from systemic lupus erythematosus patients. Our results further demonstrated that JAK-STAT pathway is activated in aged TSPCs. Interestingly, AQP1 overexpression significantly inhibited the expression of JAK-STAT targets and activators, as well as the phosphorylation of JAK2 and STAT3. Moreover, the JAK-STAT signaling pathway also has a critical role in proliferation, migration and differentiation^45,46^. Therefore, the ability of AQP1 attenuates senescence and age-related dysfunction of TSPCs through the repression of JAK-STAT signaling pathway.

In conclusion, our study demonstrated that AQP1 expression declines with age during tendon aging. AQP1 attenuated TSPCs senescence and restored the age-related reduction of self-renewal, migration and tenogenic differentiation in TSPCs. Moreover, we suggested that AQP1 modulates TSPCs senescence by regulating the JAK-STAT signaling pathway (Fig. 6l). These results provide a promising therapeutic approach for age-related tendon disorders.

## Materials and methods

### TSPCs isolation and culture

The procedures for the isolation of TSPCs from rat Achilles tendon have been well-established^7^. Briefly, rat TSPCs were isolated from 4-month-old (abbreviated as Y-TSPC), 8-month-old and 20-month-old (abbreviated as A-TSPC) male Sprague−Dawley rats (*n* = 10). The Achilles tendons were gently minced, digested with type I collagenase (3 mg/ml; Sigma-Aldrich), and passed through a 70 μm cell strainer (Becton Dickinson) to yield a single-cell suspension. The released cells were washed in phosphate buffered saline (PBS) and resuspended in Dulbecco’s modified essential medium (DMEM) containing 10% fetal bovine serum, 1% penicillin-streptomycin (all from Gibco). The isolated nucleated cells were plated at an optimal low cell density (50 nucleated cells/cm^2^) for the isolation of stem cells and cultured at 37 °C, 5% CO_2_ to form colonies. At day 7, they were trypsinized and mixed together as passage 0 (P0). Cells from P2 to P6 were used for all experiments. Medium was changed every 3 days. The clonogenicity and multilineage differentiation potential of these cells were confirmed before being used for the experiments in this study using standard assays as described previously^7^. All surgical interventions and postoperative animal care were carried out in accordance with the Guide for the Care and Use of Laboratory Animals (National Research Council) and were approved by the Animal Research Ethics Committee of Southeast University. All efforts were made to minimize the number of animals used and their suffering.

### Cell transfection

Lentivirus encoding AQP1 which labeled with GFP (LV- AQP1) and negative control lentivirus (LV-GFP) were purchased from GeneChem Corporation (Shanghai, China). 8 × 10^4^ cells were plated on six-well plates; cells at 20–30% confluence were transfected with lentivirus in accordance with the manufacturer’s recommendations. All cells were transfected by HitransG Transfection Reagent P (Genechem). The transfected cells were selected with 2 μg/ml puromycin (Beyotime Biotechnology) for 10 days to establish stably expressing cells and verified by Western blotting.

AQP1-siRNA was designed and synthetized from GenePharma (Shanghai, China); the sequences of AQP1-siRNA were as follows: CCAUGACCCUCUUCGUCUUTT (sense: 5′−3′), AAGACGAAGAGGGUCAUGGTT (antisense: 5′−3′). Transfection was performed when the cells reached 50% confluence. Cells were transfected by Lipofectamine 2000 (Invitrogen). Transfected TSPCs were used for the subsequent experiments 48 h after transfection.

### Microarray analysis

Gene expression profiles were examined by Beijing CapitalBio Corporation (Beijing, China). Briefly, poly-A containing mRNA molecules was purified from 3 μg of total RNA by using poly-T oligo-attached magnetic beads. The cleaved RNA fragments were reversely transcribed into first-strand cDNA using random hexamers, followed by second-strand cDNA synthesis using DNA Polymerase I and RNase H. The cDNA fragments were purified, end blunted, “A” tailed, and adaptor ligated. PCR was used to selectively enrich those DNA fragments that have adapter molecules on both ends and to amplify the amount of DNA in the library. The number of PCR cycles was minimized to avoid skewing the representation of the library. The library was qualified by Agilent 2100 bioanalyzer and quantified by Qubit and qPCR. The produced libraries were sequenced on the HiSeq 2500 platform. After robust multiarray average normalization, fold change threshold ≥2 and *P* value < 0.05 were recognized to be statistically significant alterations. Clustering analysis and heatmap generation were performed using Cluster3.0 software. The functional assignments were mapped onto Gene Ontology (GO). GSEA (http://software.broadinstitute.org/gsea/index.jsp) was employed to verify the biological processes in the two groups as described above^47^. NES and false discovery rate were calculated to verify the significant difference for GSEA.

### Immunofluorescence staining

For immunofluorescence staining, cultured TSPCs were fixed in 4% paraformaldehyde for 15 min at room temperature. Cells were blocked with 10% normal serum blocking solution (3% bovine serum albumin and 0.1% Triton X-100 and 0.05% Tween-20) for 2 h at room temperature. After being washed, cells were incubated overnight at 4 °C with anti-AQP1 (Proteintech) and p16^INK4A^ (Abcam), followed by a mixture of Alexa Fluor 594-conjugated secondary antibodies (Molecular Probes) was incubated 2 h at room temperature. Immunofluorescence was visualized with a Nikon Ts2R fluorescence microscope (×20 or ×40 objectives). For EdU detection, the BeyoClick™ EdU Cell Proliferation Kit with Alexa Fluor 647 was used according to the manufacturer’s protocol (Beyotime Biotechnology). Immunofluorescence was visualized with an Olympus FV1000 confocal microscope (×40 objectives).

### TSPCs migration assay

TSPCs were plated on six-well plates and grown to confluence. Then the medium was removed, and the monolayer was scratched with a sterile plastic pipette tip. The TSPCs were washed with PBS and incubated for 16 h before being imaged under an inverted microscope. The initial scratch length and scratch bridging time were measured and used for the calculation of cell velocity. Images were captured by an Olympus CKX53 inverted phase-contrast microscope (×4 objectives).

### Investigation of actin dynamics

Actin dynamics analysis was performed similarly to previous study^4^. Briefly, the young, aged and AQP1-overexpressing aged TSPCs were plated on six-well plates and incubated for 48 h. Then cells were treated with 0.4 μM Latrunculin A (Sigma-Aldrich) in a time-dependent manner (0, 5, 10, 15, 30 and 60 min). Cells were fixed in 4% paraformaldehyde and permeabilized with 0.1% Triton X-100, then the cells were stained with Alexa Flour 546 phalloidin (Thermo Scientific). Immunofluorescence was visualized with a Nikon Ts2R fluorescence microscope (×20 or ×40 objectives). For quantification of the F-actin amount, the fluorescence images were analyzed using ImageJ software (NIH). The mean fluorescence intensity was recorded.

### Western blotting

TSPCs were washed in cold PBS buffer, then the cell proteins were extracted by homogenizing the tissue in lysis buffer. The supernatant was then collected for measurement of protein concentration by BCA protein assay (Thermo Scientific). Thirty micrograms of protein was denatured, fractionated by electrophoresis on SDS-PAGE and electrophoretically transferred to a polyvinylidene fluoride (PVDF) membrane (Millipore). The blots were blocked with 5% nonfat dry milk in PBST solution, incubated with primary antibody against AQP1 (Proteintech), cyclin A2 (Proteintech), cyclin B1 (Proteintech), cyclin D1 (Bioworld), p16^INK4A^ (Abcam), JAK2 (Proteintech), p-JAK2 (Abcam), STAT3 (Proteintech), p-STAT3 (Abcam) and GAPDH (Proteintech) at 4 °C overnight. After incubating with secondary antibody, immunoreactive bands were detected by ECL reagents (Keygen Biotech). The gray value of each band was measured and data are presented as a ratio to GAPDH.

### β-galactosidase staining

The β-galactosidase (β-gal) assay was performed using the SA-β-gal staining kit (Sigma). Cells were plated on 12-well plates and incubated for 48 h. Cells were incubated with the kit’s staining mixture for 16 h at 37 °C. The percentages of β-gal-positive cells were calculated by counting 300 cells in six microscopic fields. Images were captured by an Olympus CKX53 inverted phase-contrast microscope (×4 or ×10 objectives).

### CCK-8 assay

The Cell Counting Kit-8 (CCK-8, Keygen Biotech) assay was used to measure cell proliferation. Cells were plated into 96-well culture plates at an optimal density of 3000 cells/well in 200 μl complete culture medium. The cells were observed under a microscope and the CCK-8 assay was performed at 0, 24, 48 and 72 h. Then the 10 μl CCK8 solution was added to each well and incubated for 2 h at 37 °C; the absorbance of each well was read by the microplate reader at 450 nm.

### Quantitative RT-PCR

TSPCs were harvested and homogenized for RNA extraction with the MiniBEST universal RNA extraction kit (Takara). The mRNA was reverse transcribed to cDNA by the First-Strand cDNA kit (Promega). One microliter of total cDNA of each sample was amplified in the final volume of 20 μl of reaction mixture containing Power SYBR Green PCR Master Mix (Invitrogen) and specific primers using the ABI Step One Plus system (all from Applied Biosystems). Cycling conditions were denaturation at 95 °C for 10 min, 45 cycles at 95 °C for 20 s, optimal annealing temperature for 20 s, 72 °C for 30 s, and finally at 60–95 °C with a heating rate of 0.1 °C/s. The expression of the target gene was normalized to that of the β-actin gene. Relative gene expression was calculated as fold change over control and calculated as 2^−ΔΔCt^. The primer sequences used in this study are listed in Table S3.

### Cell cycle analysis

TSPCs was cultured on 10-cm dishes in 2% FBS/DMEM for 48 h. Then cells were trypsinized and detached, washed with PBS, and then fixed in 70% ethanol overnight at 4 °C. Then, the cells were washed with PBS and incubated with RNase (Keygen biotech) and propidium iodine (Keygen Biotech) for 30 min. The percentage of cells in the three phases of the growth cycle (G1, S and G2/M phase) was measured by flow cytometry (Becton Dickinson) using Cell Quest software.

### Colony-forming unit (CFU) assays

For the CFU assay, 2, 5 and 10 cells/cm^2^ of TSPCs were plated in six-well plates respectively for 10 days in complete media. The cells were stained with 0.5% crystal violet for counting the number of cell colonies. CFU efficiency was estimated as percentage of counted colonies to the number of plated cells.

### Population doubling time (PDT) assay

Population doubling time (PDT) assay was performed as described previously^35^. PDT was calculated from the formula log2 [Nc/N0], where N0 refers to the total cell number during seeding, and Nc is the total cell number at confluence.

### Statistical analysis

All data are plotted as mean ± standard deviation (SD). Differences in mean values between groups were tested using one-way analysis of variance (ANOVA) followed by Tukey’s post-hoc multiple comparison test. *P* < 0.05 was considered statistically significant. Each experiment consisted of at least three replicates per condition. Variance was similar between the groups that were being statistically compared.

## Supplementary information

Supplementary Figure Legends

Fig S1

Fig S2

Fig S3

Fig S4

TABLE S1

TABLE S2

TABLE S3
