# Supplementary material for: AQP1 modulates tendon stem/progenitor cells senescence during tendon aging
Source: Cell Death Dis. 2020 Mar 18;11(3):193. doi: 10.1038/s41419-020-2386-3 (PMC7080760; doi:10.1038/s41419-020-2386-3)
Supplement: Supplementary file 1 — Supplementary Figure Legends [file 41419_2020_2386_MOESM1_ESM.docx]

**Supplementary Figure Legends**

Fig. S1 Microarray and gene ontology (GO) analysis of differentially expressed probe sets. (A) The transfection efficiency of LV-AQP1 was in TSPCs were investigated by western blotting. (B) Heatmap showed genes that were differentially expressed between aged and AQP1-overexpression aged TSPCs. (C-E) GO terms with the most significant p values for biological processes, cellular component and molecular function.

Fig. S2 Effects of AQP1 knockdown on young TSPCs. (A) The transfection efficiency of AQP1-siRNA was explored by Western blotting. (B) β-gal staining for the senescent cells in TSPCs. Scale bar: 100μm. (C) Quantitative analysis of β-gal positive TSPCs. (D) Western blotting for the p16^INK4A^ protein level in young and AQP1-knockdown young TSPCs. (E, F) Proliferation rate of TSPCs was measured by CCK-8 assay and PDT assay. (G) Representative scratch assay of young and AQP1-knockdown young TSPCs. Scale bar: 200 μm. (H, I) Quantification of scratch bridging time and cell velocity. (J) Relative mRNA levels of tendon-related genes (Scx, Tnmd, Bgn, Mkx, Col1A1 and Nestin) in young and AQP1-knockdown young TSPCs were investigated by qRT-PCR. Values represent the mean ± SD. *P < 0.05, significantly different from the young or AQP1-knockdown young group.

Fig. S3 Gene expression analysis of aged and AQP1-overexpressing aged TSPCs. (A) The GSEA KEGG analysis revealed the TOP 10 significant enriched signaling pathways in aged TSPCs. (B) Heatmap showed the changes in the expression of genes associated with JAK-STAT signaling pathway in aged and AQP1-overexpressing aged TSPCs.

Fig. S4 AG490 reverses the AQP1-siRNA induced senescence of TSPCs. (A) Young TSPCs were transfected with or without AQP1-siRNA and then stimulated with AG490 at 10 μM for 3h. The expressions of p-JAK2, JAK2, p-STAT3, STAT3 and p16^INK4A^ were investigated by western blotting. (B, C) β-gal staining for the senescent cells in young TSPCs upon AQP1-siRNA and/or AG490 (10 μM, 24h) treatment. Scale bars: 200μm (larger images); 100 μm (inset). Values represent the mean ± SD. *P < 0.05, significantly different from the young or AQP1-knockdown young group.
