# Supplementary figures and images for: AQP1 modulates tendon stem/progenitor cells senescence during tendon aging
Source: Cell Death Dis. 2020 Mar 18;11(3):193. doi: 10.1038/s41419-020-2386-3 (PMC7080760; doi:10.1038/s41419-020-2386-3)

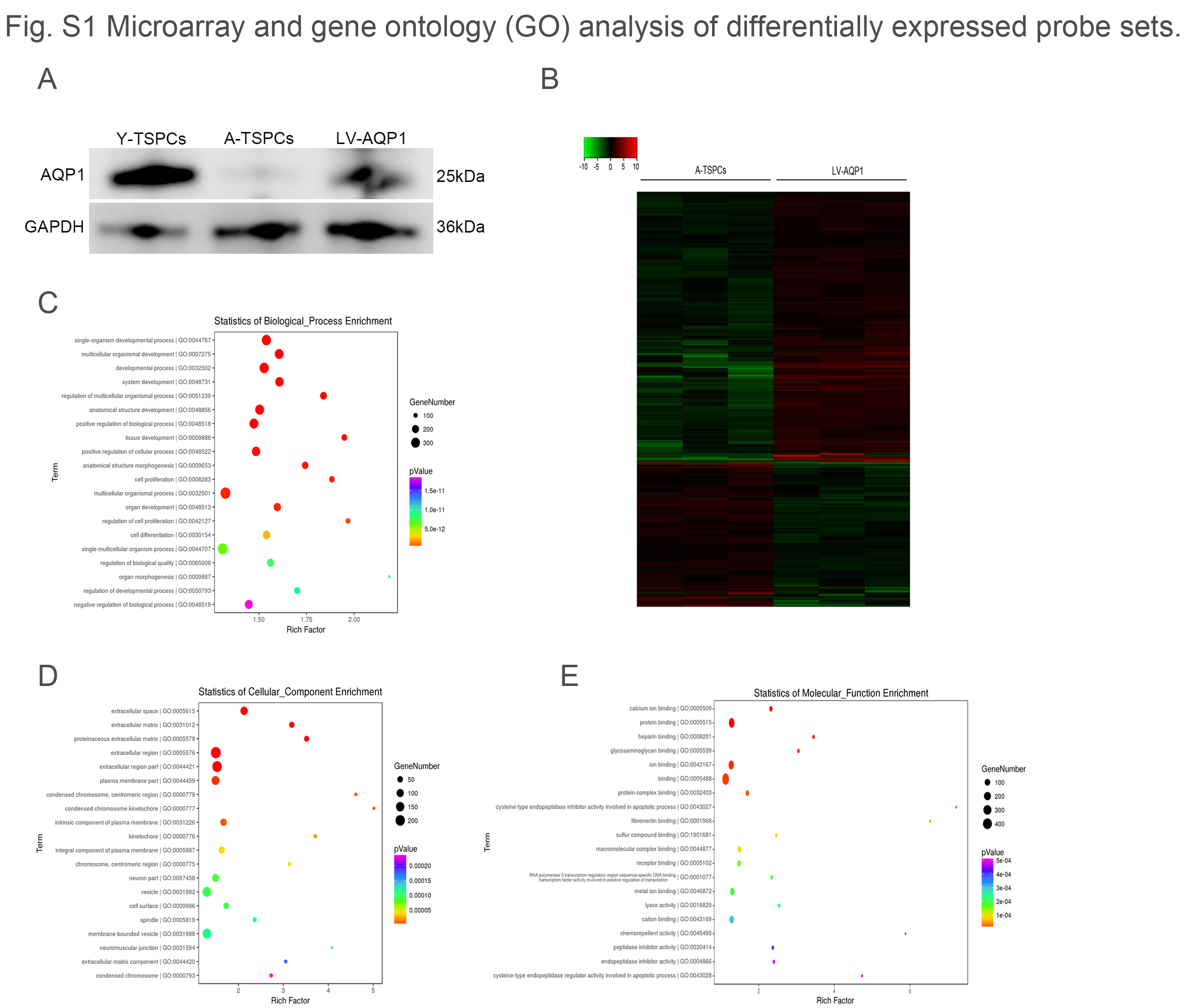

Supplement: Supplementary file 2 — Fig S1 [file 41419_2020_2386_MOESM2_ESM.tif]

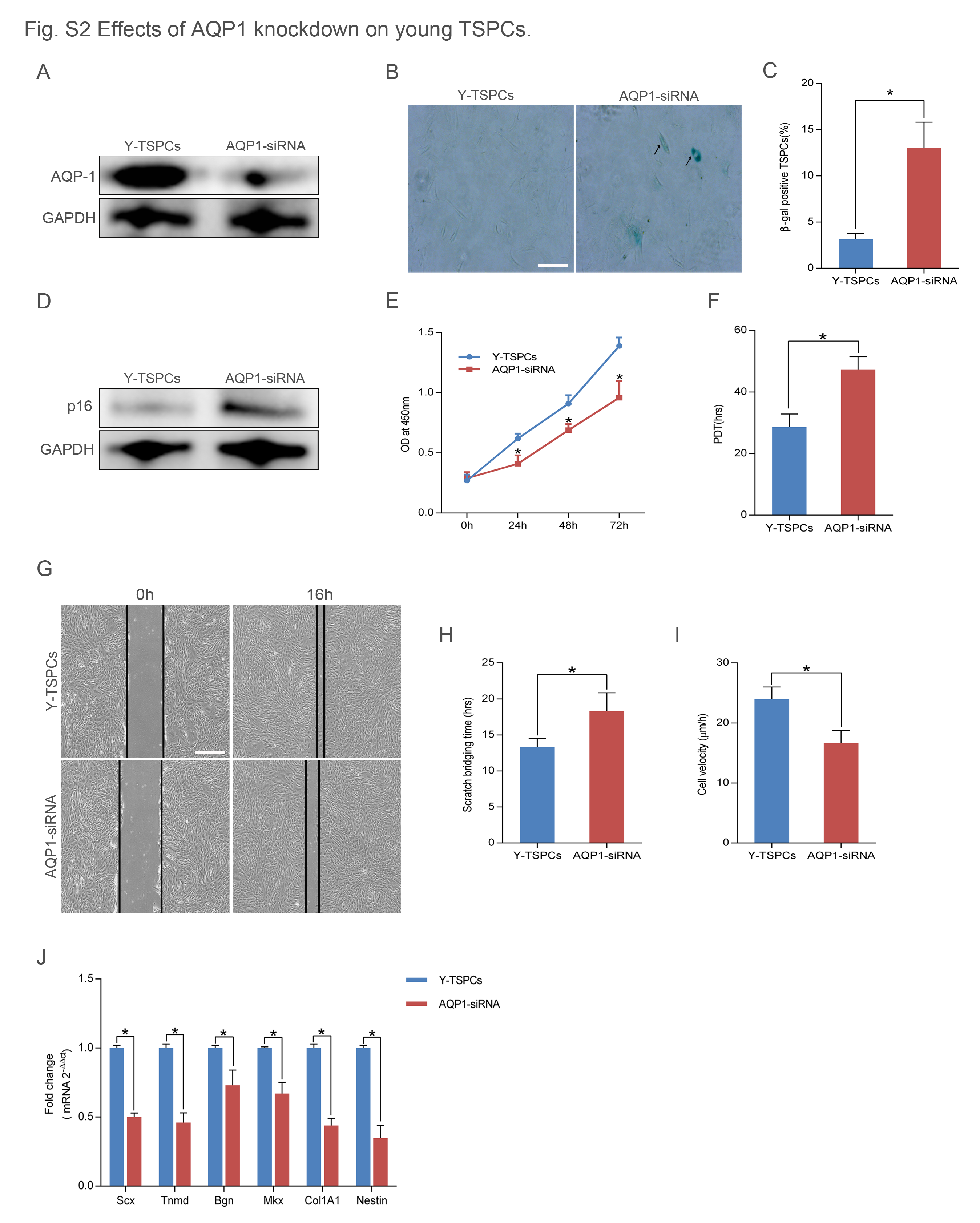

Supplement: Supplementary file 3 — Fig S2 [file 41419_2020_2386_MOESM3_ESM.tif]

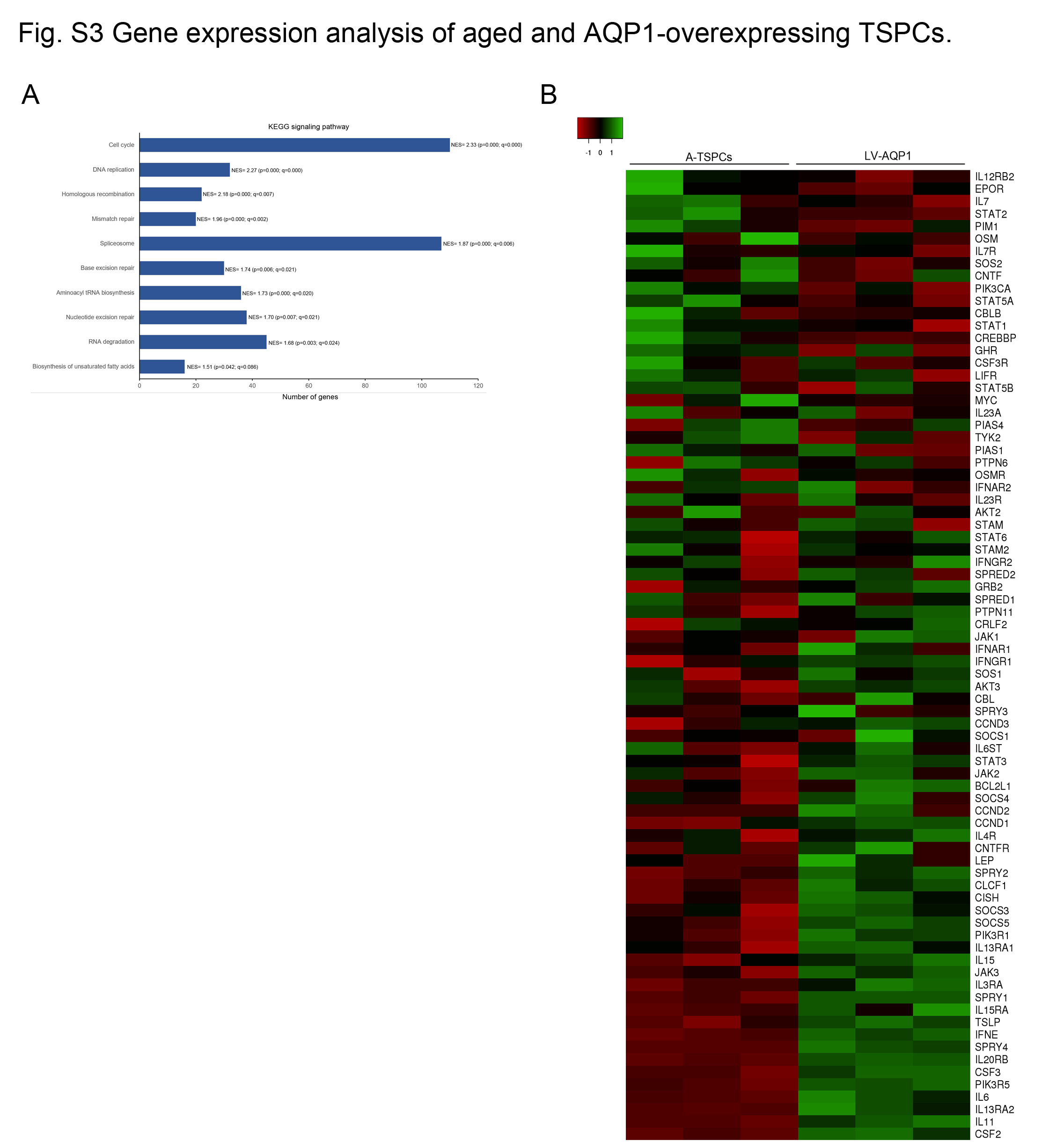

Supplement: Supplementary file 4 — Fig S3 [file 41419_2020_2386_MOESM4_ESM.tif]

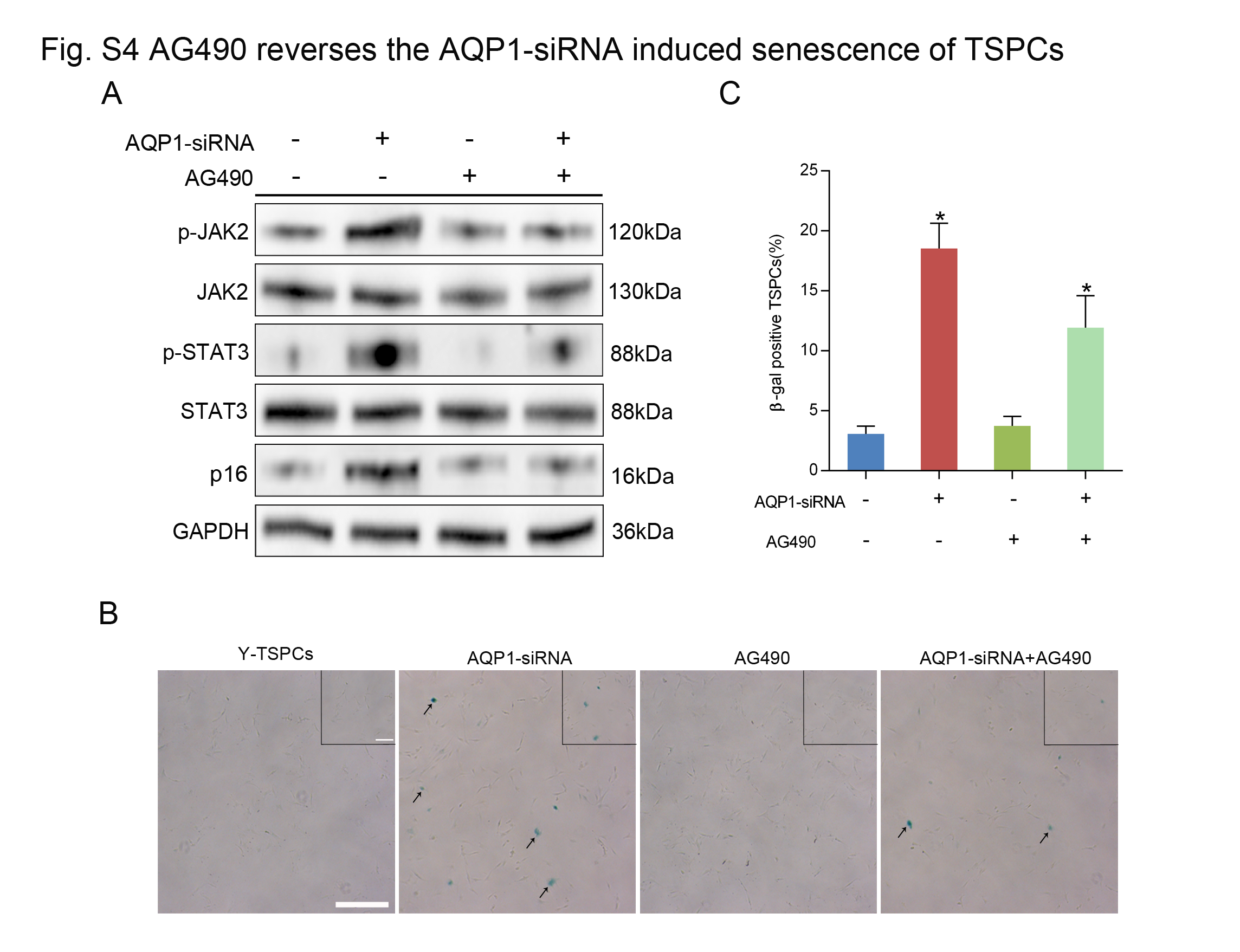

Supplement: Supplementary file 5 — Fig S4 [file 41419_2020_2386_MOESM5_ESM.tif]
