# Supplementary material for: AQP1 modulates tendon stem/progenitor cells senescence during tendon aging
Source: Cell Death Dis. 2020 Mar 18;11(3):193. doi: 10.1038/s41419-020-2386-3 (PMC7080760; doi:10.1038/s41419-020-2386-3)
Supplement: Supplementary file 6 — TABLE S1 [file 41419_2020_2386_MOESM6_ESM.docx]

| Table s1. Biological functions and genes affected after transfection | |
| --- | --- |
| Categories | Genes |
| Proliferation | Cdh13, Cd24, Tgm1, Pth1r, Hmga1, Hpse, Dchs1, Mki67, Npr3, Cdca7l, Nppb, Prkch, Fgf2, Scin, Lipg, Fgr, Prc1, Myh10, Nes, Egln3, Kcnh1,Rarres1, Met, Avp, Chst11, Rnf43, Cd274, Pgf, Cd40, Col18a1, Aldh1a2, Abo, Tek, Dll1, Tnfrsf11b, Cdc20, RGD1310335, Rerg,Ptger2, P3h2, Dusp22, Dio3, Tfap2c, Mstn, Has2, Il33, Nr4a3, Nr4a1, Hmx2, Arg1, Nox1, Syk, Sox5, Ogn, Pde1a, Wnt5a, Dhcr7, Osr2, Megf10, Kdr, Igfbp5, Cdkn2c, Odam, Six4, Igfbp2, Cd59, Cd248, Ptafr, Ptgs2, Rgn, Il6, Esm1, Racgap1, Tgm2, Lgals9, Hmgb2, Grem1, Cxcl2, Hmga2, Tk1, Il1a, Col8a2, Six1, Isl1, Cav3, Itgb3, Tfap4, Ednrb, Mycn, Duxbl1, Cask, Aldh3a1, Plac8, Wnt4, Ptprz1, Il11, Gata4, Mvd, Gucy2c, Mustn1, Mitf, Pax1, Acvrl1, Hlx, Nr3c2, Ptprf, Foxp2, Uts2r, Mmp9, Il20rb, Aspm, Spta1, Ccnb1, Vdr, Rarb, Cdk1, Htr2a, Iqgap3, Ptgir, Cebpa, Npy, Cth, Csf2, Rps6ka1, Fignl1, Txnip, Crlf1, Hoxd13, Ptges, Crip2, Ndn, Sfrp1, Cxcl10 |
