# Supplementary material for: AQP1 modulates tendon stem/progenitor cells senescence during tendon aging
Source: Cell Death Dis. 2020 Mar 18;11(3):193. doi: 10.1038/s41419-020-2386-3 (PMC7080760; doi:10.1038/s41419-020-2386-3)
Supplement: Supplementary file 7 — TABLE S2 [file 41419_2020_2386_MOESM7_ESM.docx]

| Table S2. Biological functions and genes affected after transfection | |
| --- | --- |
| Categories | Genes |
| Migration | Ptafr, Ptgs2, Cdh13, Arhgdib, Cd24, Tnn, Dchs1, Lgals9, Hmgb2, Cxcl2, Grem1, Celsr3, Fgf2, Dpep1, Dab1, Il1a, Fgr, Myh10, Six1, Isl1, Arc, Met, Itgb3, Sorbs2, Sema4g, Dcdc2, Ednrb, Ret, Cd274, Ptprz1, Wnt4, Dcn, Sema4a, Col18a1, Efna1, Mitf, Tek, Acvrl1, Cygb, Sema3b,c Sema6d, Ptprf, Prr5l, Itga7, Mmp9, Gpsm3, Aspm, Celsr2, Artn, Ccr1, Mmp10, Sema6c, Ccl3, Cmklr1, Has2, Il33, Nr4a1, Nox1, Syk, Olfm1, Mmp2, Vangl2, Adra2a, Flrt3, Wnt5a, Kdr, Igfbp5, Adamts12, Ndn, Nr2f1, Ackr3, Six4, Cd248, Cxcl10, Pla2g7, Sfrp1 |
| Actin cytoskeleton organization | Baiap2l1, Arhgdib, Tek, Cdc42ep5, Itgb3, Cav3, Acta1, Sorbs2, Gas2l3, Myh14, Racgap1, Tnik, Shroom4, Spta1, Actn2, Vangl2, Synpo, Coro2b, Wnt4, Wipf3, Scin, Il1a, Odam, Arrb1, Six4, Myh10, F11r, Epb41l3 |
