# Supplementary material for: AQP1 modulates tendon stem/progenitor cells senescence during tendon aging
Source: Cell Death Dis. 2020 Mar 18;11(3):193. doi: 10.1038/s41419-020-2386-3 (PMC7080760; doi:10.1038/s41419-020-2386-3)
Supplement: Supplementary file 8 — TABLE S3 [file 41419_2020_2386_MOESM8_ESM.docx]

TABLE 3. Primer sequences for qRT-PCR.

| Target gene | Accession No. | Forward | Reverse | | Amplicon (bp) |
| --- | --- | --- | --- | --- | --- |
| AQP1 | NM_012778.1 | 5′-GCTGTGGTGGCTGAGTTCC-3′ | 5′-TGTGACCGCTGGGTTGAG-3′ | 204 | |
| p16^INK4A^ | NM_031550.1 | 5′-CCGATACAGGTGATGATGATGG-3′ | 5′-ACCGCAAATACCGCACGAC-3′ | 253 | |
| MMP9 | NM_031055.1 | 5′-GCAAACCCTGCGTATTTCCATT-3′ | 5′-GCGATAACCATCCGAGCGAC-3′ | 83 | |
| MMP2 | NM_031054 | 5′-ACCTACACCAAGAACTTCCGACT-3′ | 5′-CAGTGTCAGTATCAGCATCAGGG-3′ | 91 | |
| DCN | NM_024129.1 | 5′-ATGGCAGTCTGGCTAATGTTCC-3′ | 5′-TGGTATGAAGGGAGGCAGAAGT-3′ | 169 | |
| p21 | NM_080782.3 | 5′-CGAGAACGGTGGAACTTTGACT-3′ | 5′-TCGGCGCTTGGAGTGATAGA-3′ | 336 | |
| p27^KIP1^ | NM_031762.3 | 5′-AATTGGGTCTCAGGCAAACTCT-3′ | 5′-TTTACGTCTGGCGTCGAAGG-3′ | 242 | |
| p53 | XM_006246594.3 | 5′-GAAGCCCTCCAAGTGTCAGC-3′ | 5′-GGCAGAACAGCTTATTGAGGGA-3′ | 220 | |
| MMP3 | NM_133523.3 | 5′-CATGAACTTGGCCACTCCCT-3′ | 5′-TGGGTACCACGAGGACATCA-3′ | 178 | |
| IL6 | NM_012589.2 | 5′-AAGAGACTTCCAGCCAGTTGCC-3′ | 5′-TGTGGGTGGTATCCTCTGTGAAG-3′ | 107 | |
| IL1B | NM_031512.2 | 5′-TGACCTGTTCTTTGAGGCTGAC-3′ | 5′-CATCATCCCACGAGTCACAGAG-3′ | 272 | |
| IL1A | NM_017019.1 | 5′-GCTAAGTTTCAATCAGCCCTTTAC-3′ | 5′-CATGATGAACTCCTGCTTGACG-3′ | 151 | |
| CXCL5 | NM_022214.1 | 5′-ATTCACCCTGCTGGCATTTCT-3′ | 5′-GCTTGTGGGTCAAGACAAACAT-3′ | 240 | |
| Scx | NM_001130508.1 | 5′ -CGAGAACACCCAGCCCAAAC-3′ | 5′-CGTCTTTCTGTCACGGTCTTTG-3 | 82 | |
| TNMD | NM_022290.1 | 5′-GACCTATGGCATGGAGCACAC-3′ | 5′- TGTTTCATCGGTGCCATTTCC-3′ | 118 | |
| BGN | NM_017087.1 | 5′-CACCTCTATGCTCTGGTCCTGG-3′ | 5′-GGTTCAAAGCCACTGTTCTCCA-3′ | 263 | |
| MKX | XM_017600733.1 | 5′-AAGGTGAGGCACAAGCGACA-3′ | 5′-ACTAGCGTCATCTGCGAGCCT-3′ | 137 | |
| COL1A1 | NM_053304.1 | 5′-AGAGGCATAAAGGGTCATCGTG-3′ | 5′-AGACCGTTGAGTCCATCTTTGC-3′ | 161 | |
| NESTIN | NM_001308239.1 | 5′-TGGAGCAGGAGAAGCAAGGTC-3′ | 5′-CAAGGGGGAAGGGAAGGATGT-3′ | 281 | |
| SOCS3 | NM_053565.1 | 5′-GGTCACCCACAGCAAGTTTCC-3′ | 5′-GCACTGGATGCGTAGGTTCTTG-3′ | 289 | |
| BCL2 | NM_016993.1 | 5′-TTGTGGCCTTCTTTGAGTTCG-3′ | 5′-GCATCCCAGCCTCCGTTAT-3′ | 151 | |
| BCL6 | NM_001107084.1 | 5′-CAGACGCACAGTGACAAACCA-3′ | 5′-CACAAATGTTACAGCGGTAGGGT-3′ | 124 | |
| PIM1 | NM_017034.1 | 5′-GCGGCGAACTCAAACTCATC-3′ | 5′-CTCAGGGACAGGCACCATCTA-3′ | 286 | |
| MYC | NM_012603.2 | 5′-AAAACCCGACAGTCACGACG-3′ | 5′-GTAGCGACCGCAACATAGGAC-3′ | 256 | |
| JUND | NM_138875.4 | 5′-CAGTACGCAGTTCCTCTACCCTAAG-3′ | 5′-AACTGCTCAGGTTGGCGTAGA-3′ | 212 | |
| CEBPD | NM_013154.2 | 5′-CGACCTCTTCAACAGCAATCAC-3′ | 5′-AAGTGGGTGGTGTGGGCTGT-3′ | 230 | |
| FOS | NM_022197.2 | 5′-ACGCTCCAAGCGGAGACAGA-3′ | 5′-TCAAGTCCAGGGAGGTCACAGA-3′ | 181 | |
| EGFR | NM_031507.1 | 5′-ATCAAAGTTCTGGGTTCAGGAGC-3′ | 5′-GACAGTGGAGGTCAGACAGATGC-3′ | 216 | |
| AR | NM_012502.1 | 5′-CTGATTCCTTTGCTGCCTTGT-3′ | 5′-ATTAGTGAAGGACCGCCAACC-3′ | 188 | |
| IL6ST | NM_001008725.3 | 5′-TGAAGTCAGAGTGGGCAACAGA-3′ | 5′-GGTGGGCTGGGTTTCACTTTAT-3′ | 193 | |
| GAPDH | NM_017008.4 | 5′-CTGGAGAAACCTGCCAAGTATG-3′ | 5′-GGTGGAAGAATGGGAGTTGCT-3′ | 138 | |
| β-actin | NM_031144 | 5′-TGCTATGTTGCCCTAGACTTCG-3′ | 5′- GTTGGCATAGAGGTCTTTACGG-3′ | 240 | |
